# Permissiveness to form pluripotent stem cells may be an evolutionarily derived characteristic in *Mus musculus*

**DOI:** 10.1038/s41598-018-32116-8

**Published:** 2018-10-02

**Authors:** Tiffany A. Garbutt, Thomas I. Konneker, Kranti Konganti, Andrew E. Hillhouse, Francis Swift-Haire, Alexis Jones, Drake Phelps, David L. Aylor, David W. Threadgill

**Affiliations:** 1 Program in Genetics, Department of Biological Science, North Carolina State University, Raleigh, NC USA; 2 Texas A&M Institute for Genome Sciences and Society, Texas A&M University, College Station, TX USA; 3 Department of Veterinary Pathobiology, Texas A&M University, College Station, TX USA; 4 Center for Human Health and the Environment, W.M. Keck Center for Behavioral Biology, and Bioinformatics Research Center, North Carolina State University, Raleigh, NC USA; 5 Department of Molecular and Cellular Medicine, Texas A&M University, College Station, TX USA

## Abstract

*Mus musculus* is the only known species from which embryonic stem cells (ESC) can be isolated under conditions requiring only leukemia inhibitory factor (LIF). Other species are non-permissive in LIF media, and form developmentally primed epiblast stem cells (EpiSC) similar to cells derived from post-implantation, egg cylinders. To evaluate whether non-permissiveness extends to induced pluripotent stem cells (iPSC), we derived iPSC from the eight founder strains of the mouse Collaborative Cross. Two strains, NOD/ShiLtJ and the WSB/EiJ, were non-permissive, consistent with the previous classification of NOD/ShiLtJ as non-permissive to ESC derivation. We determined non-permissiveness is recessive, and that non-permissive genomes do not compliment. We overcame iPSC non-permissiveness by using GSK3B and MEK inhibitors with serum, a technique we termed 2iS reprogramming. Although used for ESC derivation, GSK3B and MEK inhibitors have not been used during iPSC reprogramming because they inhibit survival of progenitor differentiated cells. iPSC derived in 2iS are more transcriptionally similar to ESC than EpiSC, indicating that 2iS reprogramming acts to overcome genetic background constraints. Finally, of species tested for ESC or iPSC derivation, only some *M. musculus* strains are permissive under LIF culture conditions suggesting that this is an evolutionarily derived characteristic in the *M. musculus* lineage.

## Introduction

Induced pluripotent stem cells (iPSC) are derived from differentiated cells that have been reprogrammed into an undifferentiated embryonic stem cell (ESC)-like state^1^. iPSCs offer a potentially unlimited source of patient-specific ESC-like cells that could be readily used for both research and therapeutics. However, the influence of genetic background on the ability of differentiated cells to be reprogrammed into iPSC has not been adequately explored. In mice, iPSC are generally derived from mouse embryonic or tail tip fibroblasts from transgenic mice of undefined or hybrid genetic backgrounds^1–8^. Few studies have used fibroblasts from inbred strains^9–11^, and even fewer studies have compared the permissiveness of differentiated cells from different inbred strains to be reprogrammed into iPSC^12^.

Derivation of pluripotent ESC from defined genetic backgrounds suggests that permissiveness to establishing pluripotency may be genetically determined^13–16^. The ability to derive naïve ESC from the inner cell masses (ICM) of pre-implantation blastocysts appears restricted to select permissive strains of mice. Examples of such permissive strains come from the 129S1/SvImJ background from which ESC were first derived. In contrast, in non-permissive strains of mice or any other species attempted, ESC cannot be derived under standard culture conditions. For non-permissive mouse strains, the earliest pluripotent stem cells that can be captured *in vitro* are epiblast stem cells (EpiSCs), derived from the post-implantation egg cylinder stage of development^17^. For most species including humans, bovine, equine, rats and deer mice, cells isolated from ICM of blastocysts or through reprogramming of differentiated cells more closely resemble the later more developmentally primed EpiSC state than the early pre-implantation ESC state in the absence of specific growth factors or pathway inhibitors^17,18^, suggesting that permissiveness may be an evolutionarily derived characteristic in *Mus musculus*.

*M. musculus* is the only known species with distinct permissive and non-permissive strains and therefore can be used to investigate the genetic basis of permissive and non-permissive states. Here we used eight genetically diverse inbred strains (five classical laboratory strains (129S1/SvImJ, NOD/ShiLtJ, A/J, C57BL/6J, and NZO/H1LtJ) and three wild-derived strains (WSB/EiJ, PWK/PhJ, and CAST/EiJ) to investigate the effect of genetic background in establishing ESC-like iPSCs. These strains represent three distinct mouse subspecies and capture about 90% most of the known genetic variation in mice^19^, a level comparable to the genetic variation found in human populations. These eight strains are also the founder strains of the recombinant inbred Collaborative Cross (CC) genetic reference population^20–22^, a genetically diverse, random, statistically powerful, and reproducible platform to investigate the genetics of mammalian traits^23,24^. An alternative, more genetically diverse outbred population called the Diversity Outbred (DO) population was also created based on these eight parental strains^25^.

The potential to create a genetically diverse iPSC panel from either of these populations would be invaluable to investigate the effects of cell based treatments in a genetically heterogeneous population. For example, the efficacy of a drug treatment could be tested *in vitro* using iPSC derived from a variety of genetic backgrounds. Although there have been no published attempts to generate iPSCs from the CC or the DO, knowledge of the inability of some of the founder strains to form ESCs suggests variability in the permissiveness of these strains. For example, sub-strains of 129, such as the 129S1/SvImJ readily form ESC^13–16^. The C57BL/6J strain is considered refractory but still permissive compared to 129 backgrounds because colony formation and germline transmission is less efficient, and cells from C57BL/6J are more likely to lose pluripotency^13,14^. Even more unfavorable is the NOD/ShiLtJ genetic background, which has been found to be non-permissive to ESC derivation in standard conditions and instead readily forms EpiSC-like cells^18^. No information is available on the ability of the five remaining CC founder strains to form ESC. In this study, we investigated all eight founder strains and found that two were non-permissive to iPSC derivation under standard conditions. The inability of two of these strains to form iPSC under standard conditions is predictive of a larger genetic background effect that could re-appear in offspring populations of the CC or the DO, thereby severely limiting the ability to conduct genetic research in cell-based systems. In this study, we characterized the non-permissive phenotype; and in response to the need to reprogram cells from diverse backgrounds, developed a novel approach to overcome the limitations imposed by non-permissive genetic backgrounds on iPSC derivation.

## Results

### NOD/ShiLtJ and WSB/EiJ are non-permissive to iPSC derivation

We evaluated the effect of genetic background on iPSC derivation using *Oct4, Klf4, Sox2*, and *Myc* in five classical laboratory strains (129S1/SvImJ, NOD/ShiLtJ, A/J, C57BL/6J, and NZO/H1LtJ) and three wild derived strains (WSB/EiJ, PWK/PhJ, and CAST/EiJ), that are the eight parental strains of the CC^19^.

**Figure 1:**
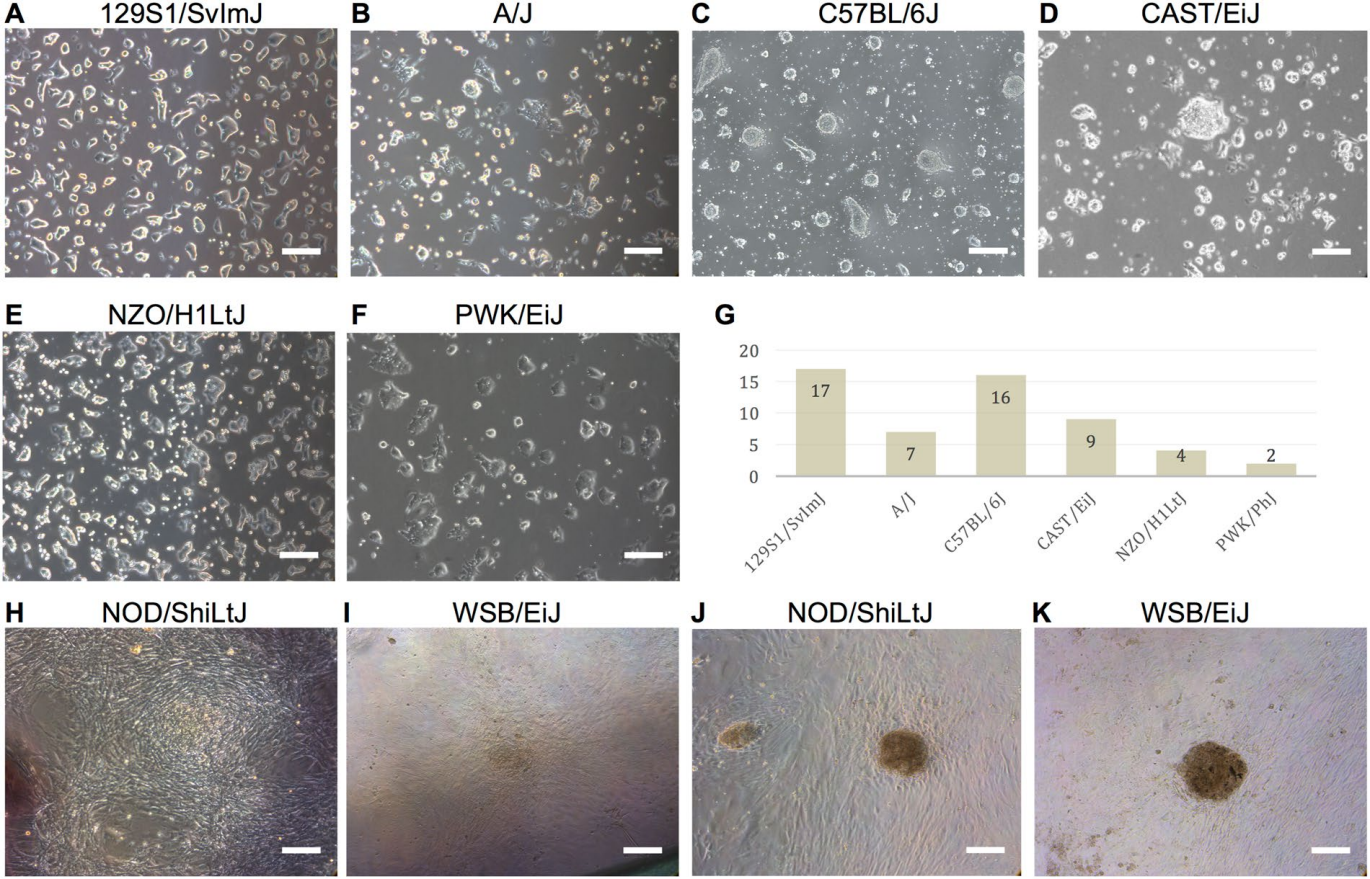
ESC-like iPSC were derived from six diverse inbred mouse strains. (**A**–**F**) The iPSC are three-dimensional dome shaped colonies that were expanded as single cells using trypsin and grown on a gelatin coated tissue culture plate. Slight differences in colony morphology were observed on gelatin. (**G**) Genetic background affected the number of iPSC derived. 129S1/SvImJ, C57BL/6J, and CAST/EiJ backgrounds produced the most iPSC. A total of 6 × 10^5^ fibroblasts were reprogrammed for each strain, with the total number of initial iPSC-like colonies subcloned from the reprogramming noted for each strain. (**H,I**) NOD/ShiLtJ and WSB/EiJ iPSC emerged as a flattened colony on the same plane as the surrounding MEF. (**J,K**) Selected NOD/ShiLtJ and WSB/EiJ iPSC were passaged manually or with collagenase type IV. The colonies retained a flattened morphology and could not be expanded even as cell clumps. All colonies showed signs of differentiation (brown color) and died within several passages. Scale bars represent 200 μm.

Six of the eight tested strains, 129S1/SvImJ, C57BL/6J, PWK/PhJ, CAST/EiJ, A/J, and NZO/H1LtJ readily formed ESC-like iPSC from mouse embryonic fibroblasts (MEF) using standard techniques (Fig. 1A–F). The iPSC colonies derived from these strains were three-dimensional and could easily be expanded from single cell suspensions using trypsin, an enzyme frequently used to passage ESC-like cells. Slight differences in colony shape were observed when the colonies were transferred from an inactivated MEF feeder layer to gelatin-coated plates, suggesting differential differentiation propensities among the strains. However, when grown on an inactivated MEF feeder layer, iPSCs from all six strains formed dome shaped colonies that maintained ESC-like in morphology over many passages. Despite being permissive, variation in the efficiency of deriving iPSC colonies was observed among the permissive strains (Fig. 1G).

In contrast, WSB/EiJ and NOD/ShiLtJ strains formed iPSC with flat morphologies (Fig. 1H,I). Following initial induction, eleven WSB/EiJ iPSCs and thirteen NOD/ShiLtJ iPSC colonies were selected for expansion, but none were capable of being passaged with trypsin and all differentiated or died within a few passages. The experiment was repeated and cells were passaged as cell clumps manually or with collagenase type IV, an enzyme used for passing EpiSC, but again all colonies differentiated and died within several passages (Fig. 1J,K). The inability of two inbred mouse strains to from ESC-like iPSC colonies suggests that genetic background plays a critical role in the success of iPSC derivation similar to ESC formation.

A previous study reported the derivation and maintenance of ESC-like iPSC from the NOD/ShiLtJ background could be achieved using ectopic expression of *Klf4* and *Myc*^9^. NOD/ShiLtJ MEF were infected with doxycycline inducible lentiviral vectors, each separately encoding one of the four iPSC transcription factors. The derivation and maintenance of NOD/ShiLtJ iPSCs was found to be dependent on continual ectopic expression of *Klf4* and *Myc*, consistent with previous reports. We suspect that we were unable to overcome the non-permissiveness of NOD/ShiLtJ and WSB/EiJ iPSC because of insufficient ectopic expression of *Klf4* and *Myc*.

### Non-permissiveness is recessive and non-complementing between non-permissive strains

**Figure 2:**
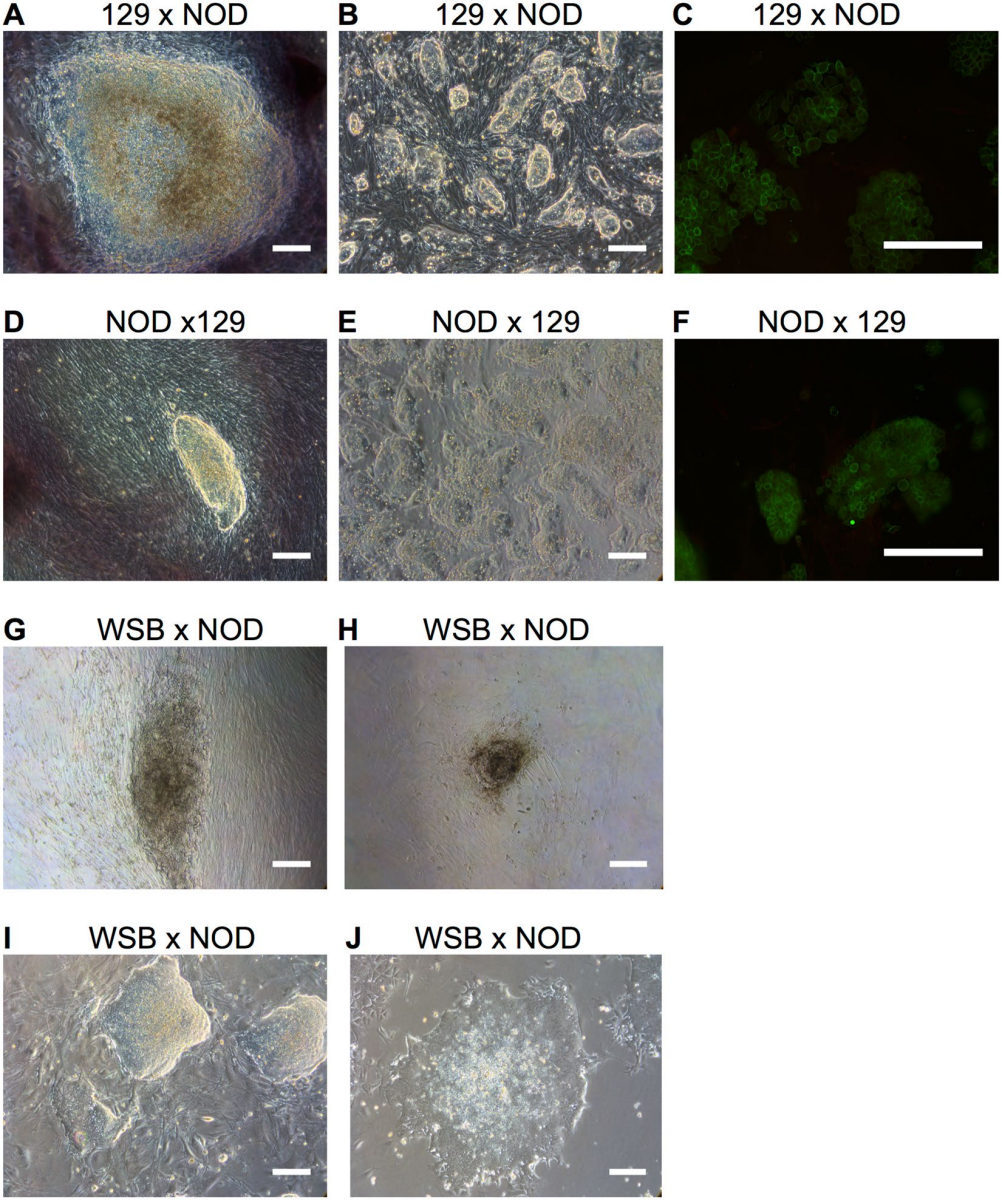
The non-permissive NOD/ShiLtJ phenotype is recessive to permissive 129S1/SvImJ. (**A,D**) Emerging iPSC were three-dimensional ESC-like colonies. (**B,E**) F1 iPSC were expanded as single cells using trypsin. (**C,F**) The cells were stained for the ESC marker PECAM1 (green) and the EpiSC maker CD40 (red). The F1 colonies stained homogenously for the ESC marker PECAM1 and did not stain for the EpiSC CD40 marker. (**G**) iPSC generated from an F1 cross between the NOD/ShiLtJ and WSB/EiJ strains were flat and showed signs of differentiation. (**H**) WSB/EiJ x NOD/ShiLtJ were passaged with trypsin. The colonies could not be expanded as single cells with trypsin and showed signs of differentiation and cell death. (**I**) WSB/EiJ x NOD/ShiLtJ iPSC developed three-dimensional dome shaped ESC-like colonies when passaged as cell clumps using collagenase type IV and grown on a MEF feeder layer. (**J**) WSB/EiJ x NOD/ShiLtJ iPSC maintained on gelatin developed flattened colonies, where individual spindle-like cells could be distinguished. Scale bars represent 200 μm.

The NOD/ShiLtJ genetic background is a well-documented non-permissive strain^9,17,26^. To characterize the inheritance pattern of the NOD/ShiLtJ non-permissive iPSC phenotype, we reprogrammed MEF from 129S1/SvImJ x NOD/ShiLtJ and NOD/ShiLtJ x 129S1/SvImJ F1 hybrids. iPSC colonies derived from the reciprocal F1 hybrids were three-dimensional, dome shaped colonies that could be readily expanded through single cell suspensions using trypsin (Fig. 2A,B,D,E), indicating that iPSC derived F1 hybrids are ESC-like in nature when cultured in ESC standard conditions. Furthermore, both reciprocal F1 hybrid iPSCs stained uniformly positive for the cell surface ESC marker PECAM1 (Fig. 2C,F). Altogether, the ability to derive ESC-like iPSC from F1 crosses between the NOD/ShiLtJ and 129S1/SvImJ strains suggests that the non-permissive iPSC phenotype is recessive to permissiveness regardless of maternal inheritance.

To investigate if the same gene or genes are responsible for the non-permissiveness of the NOD/ShiLtJ and WSB/EiJ strains, we performed an F1 complementation test using WSB/EiJ x NOD/ShiLtJ F1 hybrids. Emerging iPSC colonies derived from the F1 hybrids were flat and showed signs of early differentiation similar to that observed in both parental strains (Fig. 2G). Colonies were selected and passaged either as single cells using trypsin or as cell clumps using collagenase type IV. Reprogrammed cells differentiated and died when passaged with trypsin (Fig. 2H), while colonies that were passaged as cell clumps formed three dimensional, dome-shaped colonies on an inactivated MEF feeder layer (Fig. 2I). However, they transitioned to flattened colonies when grown on feeder-free gelatin (Fig. 2J).

Although, iPSC can be derived and maintained in culture from an F1 hybrid of WSB/EiJ and NOD/ShiLtJ strains, an iPSC phenotype similar to that of permissive strains was not observed. Unlike permissive strains, iPSC derived from WSB/EiJ and NOD/ShiLtJ F1 hybrids are intolerant to passage as single cells with trypsin and require continual growth on an inactivated feeder layer to maintain ESC-like colony formation. Altogether, our observations suggest that the WSB/EiJ and NOD/ShiLtJ backgrounds do not complement suggesting a similar genetic defect in the gene(s) controlling permissiveness.

### Non-permissiveness can be overcome using 2iS media

The use of GSK3B and MEK inhibitors in culture under serum-free conditions, commonly referred to as 2i/LIF, has been used to derive ESC from NOD/ShiLtJ and non-permissive rats^9,27,28^. The two inhibitors have also been used in combination with other inhibitors and varying culture conditions to derive iPSC from other non-permissive species such as humans, porcine, and volves^29–31^. In mice, the 2i/LIF condition has not been used to derive iPSC, but rather to complete the reprogramming process and maintain cells in an ESC-like ground state of pluripotency^9,32^. The ability to derive ESC using 2i/LIF from the non-permissive NOD/ShiLtJ background suggests that 2i/LIF may also be useful in the derivation of iPSC. Using 2i/LIF media in serum-free conditions, we attempted to reprogram MEF from NOD/ShiLtJ and WSB/EiJ backgrounds. Small colonies appeared after initial induction, but did not attach fully to the supporting MEF layer and did not expand when selected. Most notably, the MEF died in the absence of serum (Supplementary Fig. 1).

The high cell death suggests that serum may be irreplaceable during MEF reprogramming. The activation of MEK is essential to somatic cell survival and stimulates MAPK signaling, which is important for DNA synthesis and mitogenesis of differentiated cells^33,34^. Consequently, if MEK inhibition using 2i is done too early during the reprogramming process, before the somatic landscape is reset, it may result in premature cell death of somatic cells undergoing reprogramming. Indeed, others have reported that the addition of 2i/LIF in serum-free conditions to MEF three and five-days post transfection resulted in cell death and low colony survival, indicating the incompatibility of using serum-free 2i conditions during reprogramming of MEF^32^. In neural stem cells, which do not need MEK production for survival, the addition of 2i/LIF in serum-free conditions three days after initiating reprograming results in full reprogramming^32^. This suggests that if the loss of MEK activity in MEF can be overcome, then the addition of 2i/LIF shortly after infection may enhance MEF survival.

Serum does not require MEK or MAPK to stimulate DNA synthesis and mitogenesis, and contains other cytokines and growth factors that act on DNA synthesis through pathways independent of the MAPK cascade. The growth of differentiated cells in serum is not inhibited even in the presence of a MEK inhibitor. Furthermore, the MAP kinase pathway is reduced in these cells by 62% indicating that MEK is inhibited and that growth is stimulated through alternative pathways in serum^35^. We hypothesized that 2i can be used during reprogramming in serum containing conditions, referred to as 2iS, to generate ESC-like iPSC from non-permissive mouse strains.

**Figure 3:**
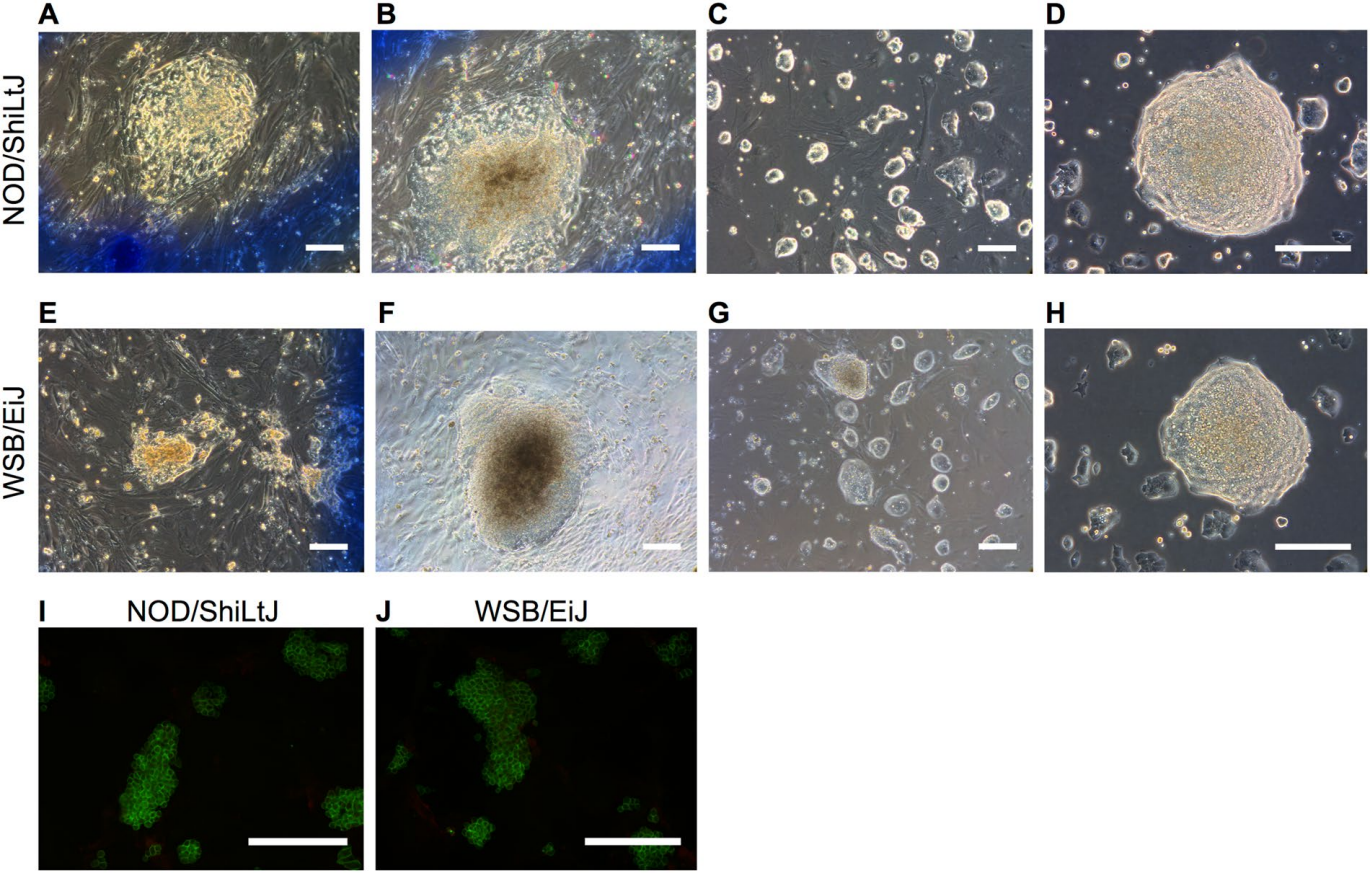
iPSC grown in 2iS were ESC-like in culture. (**A,E**) Emerging iPSC grown in 2iS were undefined colonies. (**B,F**) Prolonged culture prior to selection resulted in more defined colonies with ESC-like morphology. (**C,G**) NOD/ShiLtJ and WSB/EiJ iPSC grown in 2iS were expanded as single cells using trypsin. **(D,H)** iPSC grown in 2iS passaged with trypsin maintain an ESC-like 3D phenotype. (**I,J**) iPSC grown in 2iS stained homogenously for the ESC marker PECAM1 (green) and did not stain for the EpiSC marker CD40 (red). Scale bars represent 200 μm.

We added GSK3B and MEK inhibitors with standard ESC serum-containing conditions three days after induction of reprogramming, and maintained 2iS media throughout. Colonies began to emerge five days after 2iS addition; seven days after initiating reprogramming (Fig. 3A,E), and became more ESC-like with increased culture. By the time of colony selection, 10–20 days after initiation, colonies were three-dimensional and dome shaped (Fig. 3B,F). Selected colonies could be passaged as single cells using trypsin, as is typical for ESC (Fig. 3C,D,G,H). Twenty-nine colonies were derived from the NOD/ShiLtJ strain and thirty colonies were derived from the WSB/EiJ strain. Colonies from each strain stained homogenously for the ESC cell surface marker PECAM1 and did not stain for the EpiSC cell surface marker CD40 (Fig. 3I,J)^36^, indicating that 2iS media could overcome the non-permissive state.

### NOD/ShiLtJ Male iPSC are transcriptionally distinct from other iPSC

The sex of NOD/ShiLtJ and WSB/EiJ iPSC derived and maintained in 2iS was determined using PCR at three time points, at passages six, 10, and 16. If a colony was typed as male at an earlier passage and then typed as female at the later passage, it was determined to have lost Chromosome Y. Prior to RNA sequencing, iPSC at passage 16 or greater were MEF depleted and karyotyped according to standard protocols to determine the frequency of euploidy and to identify clones with chromosome instability^37^, which can occur in 2i conditions^38^. A minimum of 16 passages was chosen to reduce residual epigenetic differences^39^.

Male NOD/ShiLtJ colonies tended to lose Chromosome Y with increased passage, particularly between passages 10 and 16 where eight of 12 clones lost Chromosome Y after passage ten and two of 12 NOD/ShiLtJ male clones were autosomally abnormal. This is consistent with the inherent instability of Chromosome Y in ESC^40,41^, a trait that may be influenced by genetic background^40,42^. Only two NOD/ShiLtJ male clones were chromosomally normal XY males. We did not observe the same instability trend for the WSB/EiJ background.

**Figure 4:**
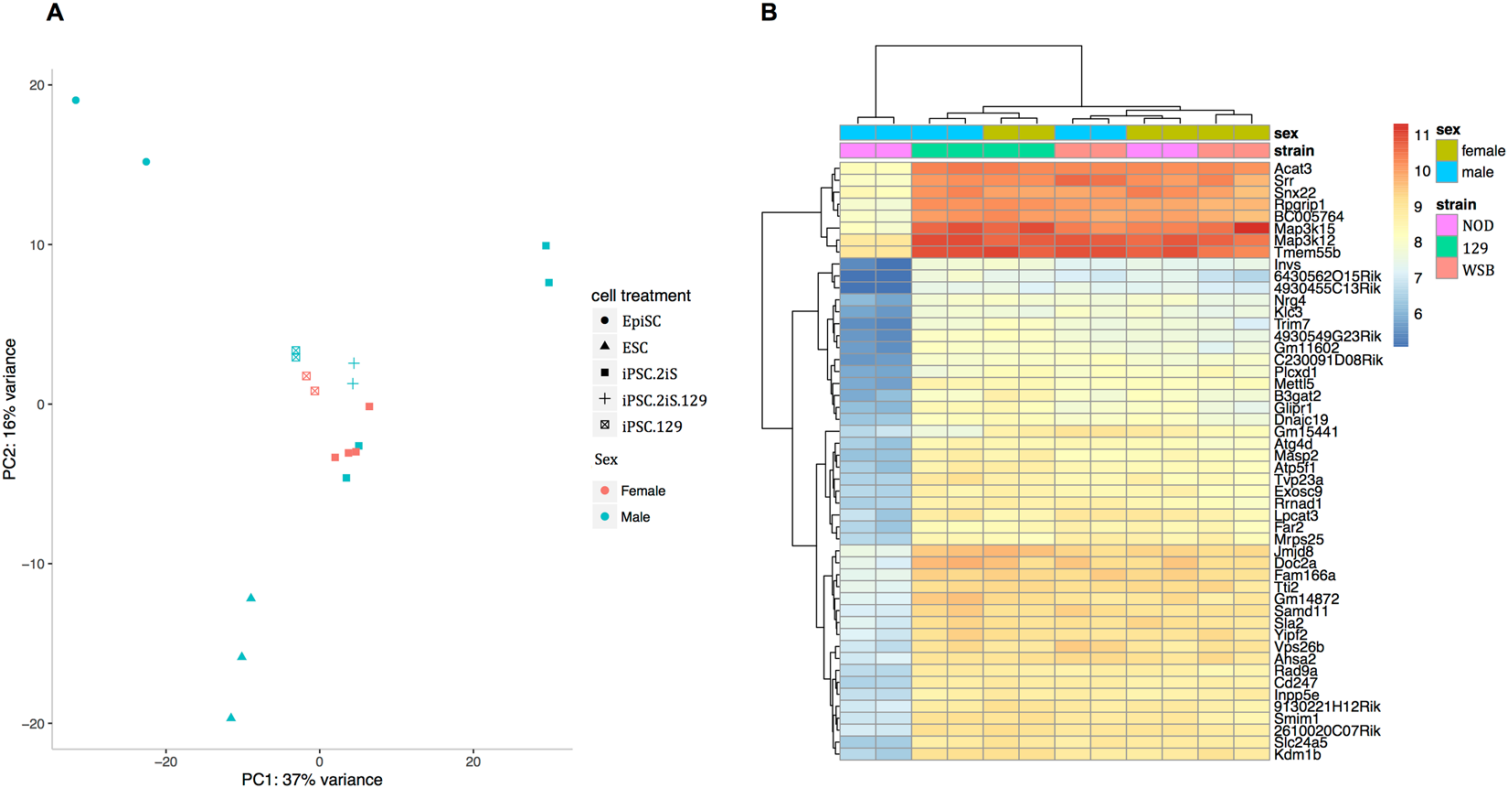
NOD/ShiLtJ male iPSC were transcriptionally distinct. (**A**) Global gene expression PCA plot of cell treatment and sex for all samples. Cells clustered according to cell state and treatment. ESC clustered together at the bottom left. EpiSC clustered together at the top left. iPSC clustered in the center of the graph and separated according to individual iPSC treatment. Male NOD/ShiLtJ iPSC grown in 2iS clustered apart from all other iPSC, ESC, and EpiSC colonies. (**B**) The transcriptional profiles of male and female iPSC from NOD/ShiLtJ, WSB/EiJ, and 129S1/SvImJ were compared. NOD/ShiLtJ male iPSC differed transcriptionally from other male and female iPSC. NOD/ShiLtJ male iPSC have a lower expression of most genes than male and female 129S1/SvImJ and WSB/EiJ strains.

Two chromosomally normal male and female iPSC clones grown in 2iS media from NOD/ShiLtJ and WSB/EiJ were processed for RNA-seq. Additionally two chromosomally normal male and female iPSC derived under standard ESC conditions from the control 129S1/SvImJ strain was also used. To assess differences between standard ESC conditions and 2iS conditions, later passages of the two male 129S1/SvImJ iPSC that were cultured in 2iS after passage 16 and maintained for eight passages were also used. Altogether, RNA-seq was performed on a total of 14 iPSC clones. RNA-seq data from iPSC was compared to RNA-seq data from true ESC derived from NOD/ShiLtJ, WSB/EiJ, and 129S1/SvImJ strains and true EpiSC from the NOD/ShiLtJ and 129S1/SvImJ strains. A global Principle Component Analysis plot revealed distinct ESC, EpiSC, and iPSC states (Fig. 4A). iPSC from the permissive 129S1/SvImJ and the non-permissive NOD/ShiLtJ and WSB/EiJ strains grown in 2iS cluster together with the exception of the two NOD/ShiLtJ male iPSC clones. Female NOD/ShiLtJ iPSC cluster with the other iPSC, suggesting the similarity of the female NOD/ShiLtJ iPSC state to other pluripotent cells.

The transcriptional sex difference between male and female NOD/ShiLtJ iPSC suggests that NOD/ShiLtJ male iPSC may have more barriers to overcome than female NOD/ShiLtJ iPSC or iPSC from other genetic backgrounds. However, no difference in the ESC cell surface marker PECAM1 was observed between male and female NOD/ShiLtJ iPSCs (Supplementary Fig. 2), suggesting that transcriptional differences between sexes do not impact colony morphology or behavior in culture. However, there may be transcriptional differences that allow for NOD/ShiLtJ males to retain Chromosome Y, but make them distinct from other iPSC. We attempted to uncover genes underlying the difference between NOD/ShiLtJ male iPSC and other iPSC by comparing male and female iPSC.

A comparison of male and female iPSC reveals a set of differentially expressed genes in the NOD/ShiLtJ males compared to the other iPSC (Fig. 4B). The expression of *Rad9a*, a gene known to increase cell viability and colony formation of ESC^43^, was lower in NOD/ShiLtJ male iPSC relative to other iPSC, suggesting decreased stem cell viability and colony formation. NOD/ShiLtJ male iPSC also had a lower expression of *Srr*, a gene whose product is known to produce, release, and covert pyruvic acid to pyruvate^44,45^, the major energy and carbon source of stem cells^46^. Due to the fact that the NOD/ShiLtJ male iPSC have an observed tendency to lose Chromosome Y and that the two remaining NOD/ShiLtJ males appear to have unique transcriptional profiles compared to the remaining iPSC, they were excluded from further analysis.

### iPSC derived with 2iS media are more similar to ESC than to EpiSC

**Figure 5:**
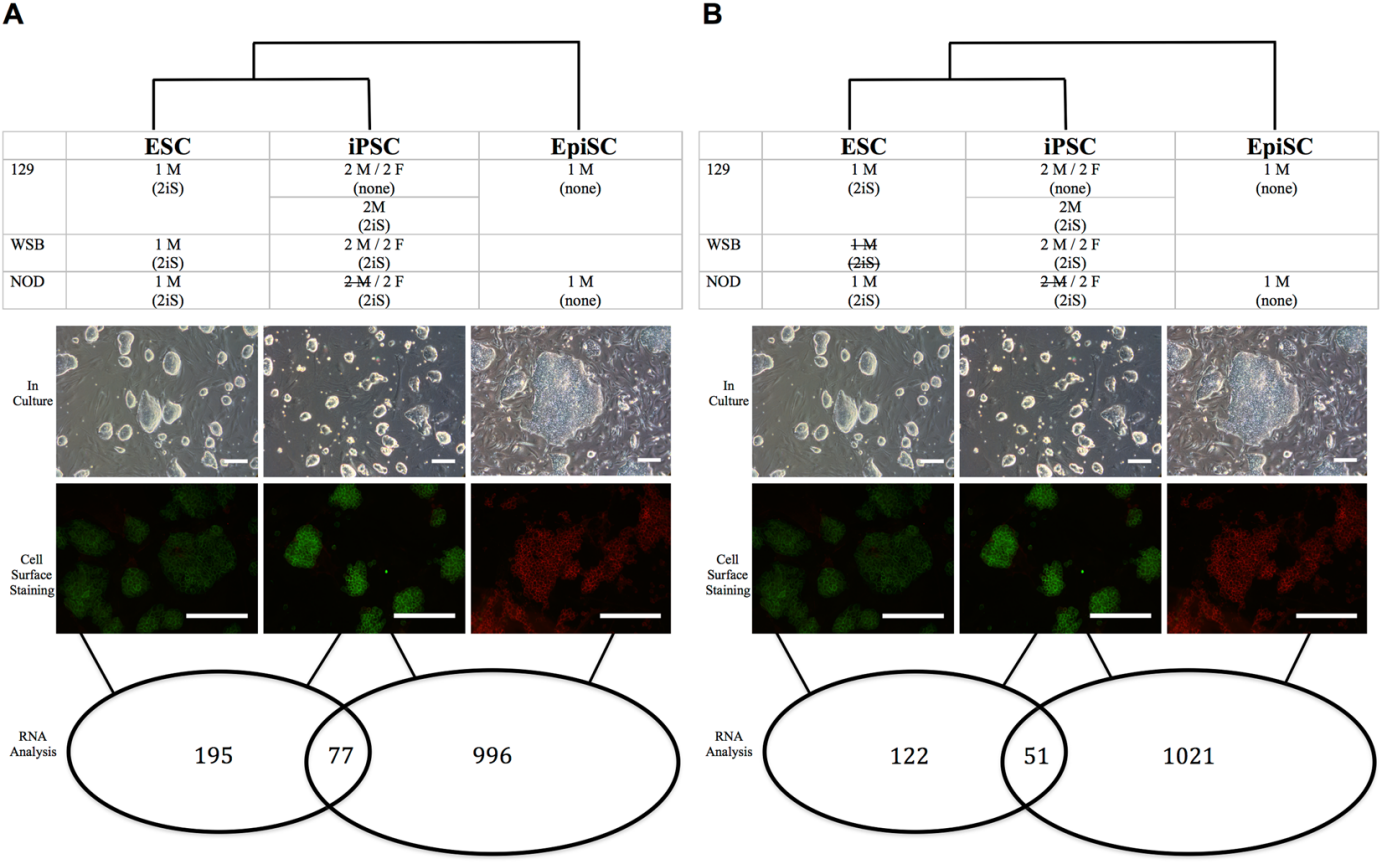
iPSC were more similar to the ESC state than to the EpiSC state. The tables list the iPSC, ESC, and EpiSC samples included in RNA sequencing. (**A**) Only the NOD/ShiLtJ male iPSC were excluded from analysis. (**B**) The NOD/ShiLtJ male iPSC and the WSB/EiJ ESC were excluded from analysis. For both tables, ESC are the first column, iPSC are the second column, and EpiSC are the third column. NOD/ShiLtJ and WSB/EiJ iPSC grown in 2iS more closely resembled ESC than EpiSC (in culture row). They stained homogeneously for the ESC marker PECAM1 (green) and did not stain for the EpiSC marker CD40 (red) (cell surface staining row). Lastly, there were fewer differentially expressed genes between iPSC and ESC than iPSC and EpiSC, indicating that iPSC-derived from NOD/ShiLtJ and WSB/EiJ grown in 2iS are transcriptionally closer to the ESC cell state (RNA analysis row). Scale bars represent 200 μm.

Because non-permissive strains readily form EpiSC instead of ESC, we investigated whether iPSC derived with 2iS are transcriptionally closer to the ESC state than to the EpiSC state. The transcriptional profile of all iPSC derived in this study were compared to the transcriptional profiles of ESC and EpiSC. iPSC derived from the NOD/ShiLtJ and WSB/EiJ backgrounds using the 2iS reprogramming technique cluster with iPSC from the permissive 129S1/SvImJ background (Fig. 5A), indicating that all iPSC derived with 2iS are transcriptionally similar with the exception of the two NOD/ShiLtJ male clones. We observed substantially more differentially expressed genes when the iPSC were compared to EpiSC.

There is the possibility that the WSB/EiJ background may drive this difference, as there is a WSB/EiJ ESC, but no WSB/EiJ EpiSC in the comparisons. To address this, we excluded the WSB/EiJ ESC and performed each analysis again (Fig. 5B). Although the number of differentially expressed genes in the iPSC versus ESC comparison decreased, fewer differentially expressed genes are still observed in the iPSC versus ESC comparison than in the iPSC versus EpiSC comparison, supporting that the derived iPSC are closer to the ESC state than to the EpiSC state.

The ESC and EpiSC used in our analyses were all male, due to the fact that male stem cells are generally used and more available than female stem cells. These male ESC and EpiSC were compared to our panel of male and female iPSC, thereby creating a sex unbalance. However, this unbalance is maintained in both comparisons with male and female iPSC being compared to only male ESC or male EpiSC. Any female specific genes would be found in both comparisons and would consequently fall within the overlapping region of differentially expressed genes. Therefore, this sex unbalance is not expected to pose problems for detecting and comparing the number of non-overlapping differentially expressed genes in each comparison.

Some differences in culture conditions were unavoidable and were directly due to the culture requirements of different cell types. For example, ESC require different growth conditions than EpiSC. Differences in iPSC culture conditions were due to the culture requirements of permissive and non-permissive strains, with NOD/ShiLtJ and WSB/EiJ requiring 2iS culture conditions for derivation. Consequently, we sought to identify transcriptional differences between iPSC cultured in 2iS and iPSC cultured in standard ESC media. Because stable iPSC could not be derived without 2iS from the non-permissive NOD/ShiLtJ and WSB/EiJ backgrounds, we used the permissive 129S1/SvImJ strain. 2iS media was added after passage 16 and maintained for eight passages to two 129S1/SvImJ male iPSC previously derived in standard ESC/LIF conditions. The cells were then MEF depleted before harvesting for RNA-seq. We compared the transcriptional profile of the two 129S1/SvImJ males in standard ESC/LIF media to their transcriptional state in 2iS media. Only 39 differentially expressed genes were detected between the two conditions, indicating little difference between culture conditions in the permissive 129S1/SvImJ background (Supplementary Fig. 3).

## Discussion

Here we tested the ability of eight diverse, inbred mouse strains that are founders of the CC population to derive ESC-like iPSC and found that the NOD/ShiLtJ and WSB/LtJ strains are non-permissive in standard conditions. Previous knowledge indicates that the NOD/ShiLtJ background is non-permissive to ESC formation and instead forms cells that more closely resemble the EpiSC state of mouse development^17,18^. We conclude that initial colonies formed by the NOD/ShiLtJ and WSB/EiJ strains were EpiSC-like in nature, characterized by a flattened morphology and inability to be maintained in standard ESC culture conditions. Thus, we aimed to derive ESC-like iPSC from the NOD/ShiLtJ and WSB/EiJ non-permissive strains.

Inhibitors blocking GSK3B and MEK have been found to overcome non-permissiveness in ESC derivation from the NOD/ShiLtJ mouse strain and non-permissive rat species^9,27,28^. However, GSK3B and MEK inhibitors are typically used in serum-free LIF conditions that are incompatible with fibroblast viability and reprogramming^32^. Furthermore 2i/LIF cannot be added post reprogramming, as it is ineffective at eliciting a change from the EpiSC state to the ESC state^47^. To capitalize on the ability of GSK3B and MEK inhibitors to overcome non-permissiveness, but maintain fibroblast viability during reprogramming, we added the two inhibitors with serum (2iS) three days into reprogramming and maintained 2iS conditions throughout colony selection and expansion. The resultant iPSC colonies were three-dimensional with ESC-like in morphology, could easily be expanded as single cells, and stained positive for the ESC maker PECAM1.

NOD/ShiLtJ male iPSC were prone to losing Chromosome Y with prolonged culture in 2iS. Prolonged culture in 2i has been linked to increased chromosomal abnormalities^38^, but such changes were not observed in the WSB/EiJ strain, suggesting that the chromosomal instability of the NOD/ShiLtJ Chromosome Y is affected by genetic background. ESC lines have a tendency to lose Chromosome Y at a frequency of 2–2.9%^40,41^. However, we observed eight out of twelve NOD/ShiLtJ male iPSC lines lost Chromosome Y (66.7%). The two NOD/ShiLtJ males that retained Chromosome Y may have other abnormalities as they are transcriptionally distinct from the other iPSC and have decreased expression of *Rad9a*; ESC with decreased *Rad9a* have higher levels of catenated mitotic spreads^48^.

Because non-permissive strains typically produce EpiSC, we compared the transcriptional profile of the iPSC to ESC and EpiSC states. The iPSC were closer transcriptionally to the ESC state than to the EpiSC state, providing evidence that 2iS is successful in deriving ESC-like iPSC from non-permissive strains. To our knowledge this is the first study to derive ESC-like iPSC from MEF of non-permissive strains by using addition of serum with GSK3B and MEK inhibitors during the reprogramming process. This discovery is applicable to deriving ESC from more differentiated cells, such as fibroblasts, that require MEK for survival and may be helpful for other non-permissive genetic backgrounds and species.

**Figure 6:**
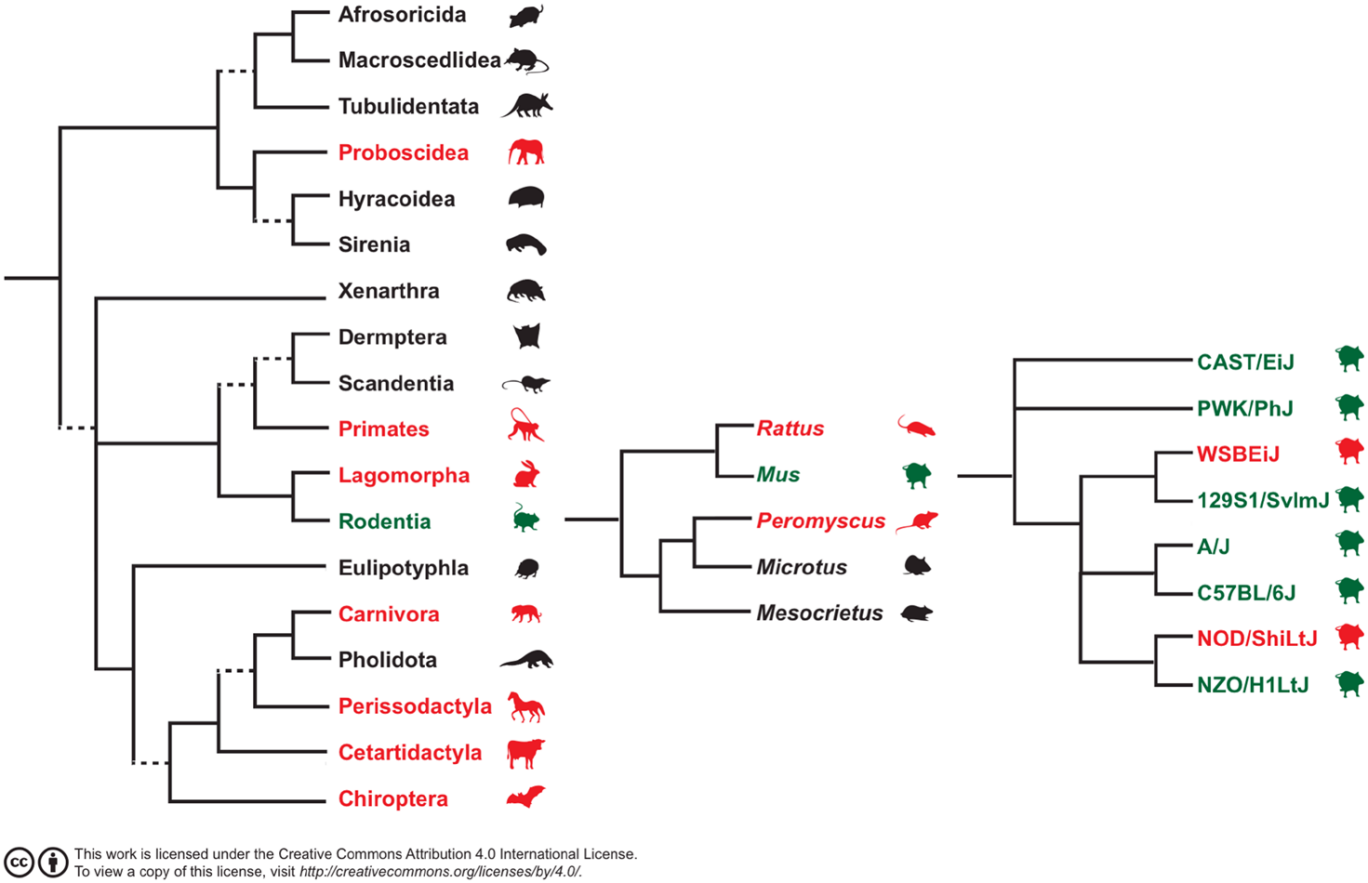
Cladogram of eutherian mammal orders with rodent genus groups and the eight *M. musculus* lines. Groups that have representatives from which ESC or iPSC have been derived are noted in color. Red, based on culture conditions are non-permissive to simple derivation; green, permissive to simple pluripotent stem cell derivation.

Genetic background affects iPSC derivation with some genetic backgrounds readily forming ESC-like iPSC and others resistant to iPSC formation under standard conditions. This is similar to what is observed for ESC derivation under standard conditions for permissive and non-permissive strains. All other species, including humans, form primed stem cells that are more similar to mouse EpiSC-like cells using standard ESC/LIF conditions suggesting that the permissiveness in *Mus musculus* may be an evolutionarily derived characteristic not present in other eutherian mammals (Fig. 6). This study reports the novel use of 2iS media during reprogramming to derive ESC-like iPSC from MEF of non-permissive mouse strains and shows the influence of genetic background on transcriptional differences among ESC-like iPSC.

## Materials and Methods

### Cell lines

A range of 2 to 8 embryos were collected from one arm of the uterus of a pregnant dam 12.5–14.5 dpc for each inbred strain 129S1/SvImJ, C57BL/6J, PWK/PhJ, CAST/EiJ, A/J, NOD/ShiLtJ, WSB/EiJ, NZO/H1LtJ. Differentiated regions, such as the head, limbs, tail, heart, and kidney were removed from each embryo. Embryos from the same litter were then minced, pooled together, and treated with trypsin to break down cellular mass into single cell fibroblasts. The single cell suspension was then plated into flasks and allowed to expand to confluency. Confluent populations were frozen slowly using Life Technologies Recovery Cell Culture Freezing Medium, liquid at −80 C for 1 to 2 weeks and then transferred to liquid nitrogen for long-term storage. The same procedure was followed for the F1 hybrids used in this study. All animal studies were approved by the NC State University Institutional Animal Care and Use Committee.

NOD/ShiLtJ, WSB/EiJ and 129S1/SvImJ ESCs used for RNA sequencing were provided by Dr. Laura Reinholdt (The Jackson Laboratory, JAX). These ESC were derived using standard JAX protocols and maintained in ESC/LIF serum conditions with sodium pyruvate and supplemented with GSK3B and MEK inhibitors^49^. RNA was extracted from flash frozen cell pellets.

The NOD/ShiLtJ EpiSC used for RNA sequencing was provided by Dr. Ludovic Vallier^17^. The 129 EpiSC (FT129/4: 129/Sv background) used for RNA sequencing was supplied by Dr. Alice Jouneau^50^. Both EpiSCs were maintained as a previously described^17,50^. RNA was extracted from flash frozen cell pellets.

### Lentiviral reprogramming and colony expansion

Lentiviral reprogramming was performed using the STEMCCA Cre-Excisable Constitutive Polycistronic (OKSM) Lentivirus Reprogramming Kit from Millipore as per the manufacturer’s instructions. The same procedure was followed for all genetic backgrounds and reprogramming experiments. Genes for the four transcription factors, *Oct4*, *Sox2*, *Myc*, and *Klf4* integrate randomly into the genome, are constitutively expressed, and can be removed with the addition of an adenovirus-encoded cre-recombinase that acts on flanking loxp sites. However, the ectopically expressed OKSM factors were not removed in the present study.

Primary MEF at passage three were seeded at a density of 1 × 10^5^ cells onto a 0.1% gelatin coated six-well plate and allowed to expand in MEF expansion media for 24 hours in a 37C, 5% CO2 incubator. A total of 6 × 10^5^ fibroblasts (1 × 10^5^ cells per well on a six well plate) were reprogrammed for each strain. The MEF expansion media was replaced after 24 hours, polybrene transfection agent, and the virus were added at the optimal multiplicity of infection of 20. Standard procedures were followed to ensure even viral distribution and plates were incubated at 37 C overnight. After 24 hours the media was replaced with ESC media and was renewed every other day. Mouse iPSC colonies began to emerge between days 7 to 10 post transfection and were allowed to expand until the colony fit into the frame of a 10x magnification view around day 12 to 20 post transfection. Individual colonies were selected by manual dislodging and pipetting using a 200 ul wide orifice pipette and transferred to a 0.05% trypsin solution to digest the colony into single cells. The cell solution was then transferred to a 24 well gelatin coated plate containing a feeder layer of mytomycin inactivated MEF. The colonies were allowed to expand and were split every two to three days until they were confluent on a 10 cm tissue culture dish.

### iPSC selection conditions

#### 2i media

2i media containing LIF (Millipore) was added two days after virus addition and replaced every two days. Selected cells were passaged manually or as cell clumps using collagenase type IV.

#### 2iS media

One day after the virus was added, the media was removed and fresh ESC/LIF media was added to the wells. Three days after virus addition, the ESC/LIF media was removed and 2iS media was added. The media was replaced every other day. 2iS media was made just before use by adding the thawed inhibitors to ESC/LIF media. Some selected cells were initially passaged manually or as cell clumps using collagenase type IV and some selected colonies were passaged using 0.05% trypsin.

### Medium and culture conditions

#### Mouse Embryonic Fibroblast Medium

Mouse embryonic fibroblasts were grown and maintained in MEF Media. The composition of MEF media was DMEM GlutaMax (Life Technologies), 1% Penicillin/Streptomycin Solution (100X) (Life Technologies), and 10% Fetal Bovine Serum (Atlanta Biologicals). Mouse embryonic fibroblasts were passaged at least once a week using 0.25% Trypsin-EDTA (1x) with phenol red (Life Technologies).

#### Embryonic Stem Cell (ESC/LIF) Medium

The composition of ESC/LIF media was DMEM GlutaMax (Life Technologies), 1% Penicillin / Streptomycin Solution (100X) (Life Technologies), 15% ES Qualified Fetal Bovine Serum (Atlanta Biologicals), beta-mercaptoethanol (1000x) (Life Technologies), 1% Non-essential Amino Acid (NEAA) solution (100x) (Life Technologies), LIF (10 million units/1 ml) (Millipore). Cells were passaged using 0.05% Trypsin-EDTA (1X) with phenol red every two to three days.

#### 2i/LIF Medium

*2i/LIF media*: Serum free 2iS/LIF medium was purchased as ESGRO-2iMedium from Millipore.

#### 2iS Medium

2iS media was comprised of ESC/LIF media and supplemented with 3 uM of GSK3B inhibitor CHIR99201 and 1 uM of MEK inhibitor PD0325901. 2iS media was made just before use, by adding the thawed inhibitors to ESC/LIF media.

### RNA sequencing

Cells were flash frozen using liquid nitrogen as cell pellets and stored at −80 until RNA and/or DNA extraction could be performed. All RNA extractions were done using the Promega Maxwell LEV SimplyRNA extraction kit. cDNA, library prep, and RNA sequencing was performed by the Texas A&M Institute for Genome Sciences & Society. Extracted RNA was run on a Bioanalyzer (Agilent) to confirm sample integrity. cDNA libraries were created using the TruSeq Stranded mRNA library prep kit (Illumina). Samples were pooled and run on a 150 × 150 high output NextSeq500, generating 800 million paired end reads (approximately 32 million reads/sample).

### RNAseq analysis

A total of 453 million reads were checked for any adapter sequences and to trim any low-quality bases using Trimmomatic resulting in approximately 285 million filtered reads (63%) out of which a total of 229 million filtered reads (approximately 80%) mapped to the GRcm38/mm10 assembly^51^. Read mapping was performed using TopHat version 2.1.1^52^. HTSeq was used to generate raw read counts per gene using intersection-nonempty parameter to account for ambiguous read mappings^53^. Differential gene expression tests were then performed used DESeq2 following recommended guidelines by the authors^54^. Plots were generated using R programming language. The resulting gene expression values were uploaded to the Ingenuity Pathways Analysis (IPA) program (QIAGEN; Application Build 261899, Content Version 18030641) for gene function and biological pathway analysis. Additionally, the compare feature of IPA was used to identify the number of unique and overlapping differentially expressed genes between the iPSC vs. ESC and the iPSC vs. EpiSC comparisons.

The heatmap plots are shown in r-log, which is a moderated log-2 transformation of the data. The data has had a variance-stabilizing transformation applied before log-2 transformation. The variance-stabilizing transformation lowers the estimated fold change for genes with a low overall expression value, making these changes appear less drastic in plots.

### Sex PCR

Cells were flash frozen using liquid nitrogen as cell pellets and stored at −80 until DNA extraction could be performed. DNA was extracted using the Maxwell SEV Cell DNA extraction kit (Promega) from individually selected colonies. The sex of individual expanded colonies was determined using polymerase chain reaction (PCR) of the highly homologous SMC gene found on Chromosome X (*Smcx* = kdm5c) and Chromosome Y (*Smcy* = Kdm5d). Primer sequences used: SMCX-1: CCGCTGCCAAATTCTTTGG and SMC4-1: TGAAGCTTTTGGCTTTGAG. Thermocycler conditions: 35 cycles of: 95 C for 30 sec, 60 C for 30 sec, 72 C for 1 minute. PCR products were run on a 2.5% agarose gel. Due to homozygosity, a single *Smcx* band at 350 bp identified females. The mouse *Smcy* gene is missing an intron found in *Smcx* resulting in s shorter PCR product. Due to the intron difference, two bands at 350 and 300 base pairs were used to identify males^55^.

### MEF depletion

After expansion on a 10 cm tissue culture plate to passage 16 or greater, the colonies were MEF depleted to ensure only stem cells were collected for RNA or DNA extraction. The media was aspirated; the plates were washed with PBS, and treated with 0.05% trypsin. The trypsin was inactivated with the addition of media and all the cells were transferred to an un-gelatinized petri dish. The dish was incubated at 37 C for 1 hour, after which MEF bound to the petri dish and stem cells continued to float in the media. The stem cells were collected and plated onto a gelatinized tissue culture plate. The process was repeated as necessary to further deplete any remaining MEF.

### Karyotyping

Previously documented data support the idea that passaging iPSCs to passage 16 or greater eliminates transcriptional and epigenetic differences among iPSC lines^39^. Furthermore, emerging anecdotal evidence suggest that prolonged culture with the GSK3β and MEK inhibitors may promote karyotypic instability^38^. Consequently, we passaged all iPSC including those derived and maintained in 2iS media for 16 passages or greater. The iPSCs were then MEF depleted and split into four 10 cm tissue culture plates, one for DNA collection, one for RNA collection, one for storage, and one for karyotyping. If needed a second MEF depletion was performed before collection. Karyotyping was performed according to established protocols^56^. DAPI DNA stain was used for easier visualization. Images of twenty to forty spreads in widely separated fields were collected. The number of chromosomes in each spread was counted and then recounted by a separate impartial counter. If a discrepancy between the first two counts was recorded, the number of chromosomes was counted by a third impartial counter. Lines with 39 chromosomes in half of the counted spreads and a sex PCR profile of female were determined to be XO. Lines with 39 chromosomes in half of the counted spreads and a sex PCR profile of male were determined to have some other chromosome abnormality. iPSC lines with a minimum of 70% of spreads containing 40 chromosomes were considered karyotypically normal.

### Immunofluorescence staining

Square coverslips (18 × 18 mm, 1 oz, 0.16–0.19 mm thickness) were sterilized by in 70% ethanol and allowed to air dry under UV light for 1 hour. Following sterilization, one coverslip was placed flat into each well on a six well tissue culture plate. A 0.1% gelatin coating solution was placed in each six well, allowed to completely cover the surface of the well and coverslip, and incubated for 5 minutes at room temperature. Following incubation the remaining gelatin was aspirated.

A vial of mitomyicin treated inactivated primary MEFs from Millipore was thawed using the slow freeze/fast thaw technique for thawing cells. A volume of 10 ul was used to count the number of MEFs using a Cell Countess (Invitrogen). The cells were diluted and distributed in MEF media to the appropriate volume to cover a six well plate and incubated overnight at 37 C. The following day the media was replaced with fresh MEF media until stem cells were added to the prepared MEF plates. When stem cells were added to the MEF plates the media was changed to ESC/LIF media.

After two to three days of growth, when stem cells cells have reached the desired density, the culture medium was aspirated and the cells were washed twice in PBS. The cells were fixed in 2% paraformaldehyde and incubated at room temperature for 20 minutes. After incubation the cells were washed twice with PBS. Non-specific staining was blocked by adding 1 ml of blocking buffer (1% BSA in PBS) and incubating for one hour at room temperature after which the blocking buffer was removed. PECAM1 monoclonal primary antibody (BD Biosciences 553370) was diluted 1:400 or CD40 polyclonal primary antibody (R&D Systems AF440) was diluted 1:500 in dilution buffer (0.1% BSA in PBS) before adding to the wells. Primary antibodies were incubated at 4C overnight.

The following day the primary antibody solution was removed and the cells were washed three times in PBS. The secondary polyclonal antibody (Alexa Flour 488-labeled donkey anti-rat IgG, Jackson Antibodies 712-545-153 for PECAM1 or Alexa Flour 546-labeled donkey anti-goat IgG, Life Tech A11056 for CD40) was diluted 1:400 in dilution buffer, added to the wells, and incubated in the dark at room temperature for one hour. Following incubation, the secondary antibody solution was removed and the cells were washed three times in PBS. The coverslips were then removed with a fine tip tweezers from the six-well plate and air dried for approximately 30 minutes. The coverslips were then placed, cells down in one drop of VECTASHIELD HardSet Antifade Mounting Medium with DAPI on a 25 × 75 × 1 mm rectangle slide. The coverslips were allowed to adhere to the slide for 15 minutes and stored at 4 C until visualization. Visualization and image capture were performed one to two days following immunofluorescence staining using a Lieca DM 5500B fluorescence microscope.

### Compliance

All methods were performed in accordance with the relevant research guidelines and regulations.

## Electronic supplementary material

Supplementary Figures

## Data Availability

The RNAseq data used in the analysis is available through the NCBI Gene Expression Omnibus (Accession Number GSE110579).
