# Supplementary material for: Permissiveness to form pluripotent stem cells may be an evolutionarily derived characteristic in Mus musculus
Source: Sci Rep. 2018 Oct 2;8:14706. doi: 10.1038/s41598-018-32116-8 (PMC6168588; doi:10.1038/s41598-018-32116-8)
Supplement: Supplementary file 1 — Supplementary Figures [file 41598_2018_32116_MOESM1_ESM.pdf]

Permissiveness to form pluripotent stem cells may be an evolutionarily derived characteristic in *Mus musculus*

Tiffany A. Garbutt, Thomas I. Konneker, Kranti Konganti, Andrew E. Hillhouse, Francis Swift-Haire, Alexis Jones, Drake Phelps, David L. Aylor, David W. Threadgill

Supplemental Information

NOD/LtJ

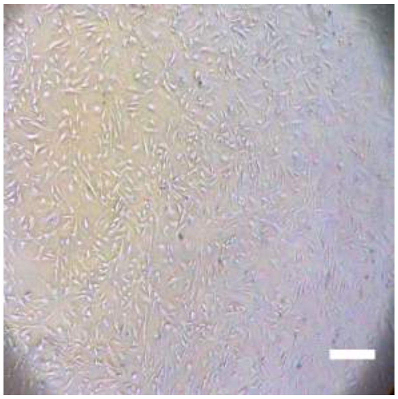

A. day before virus

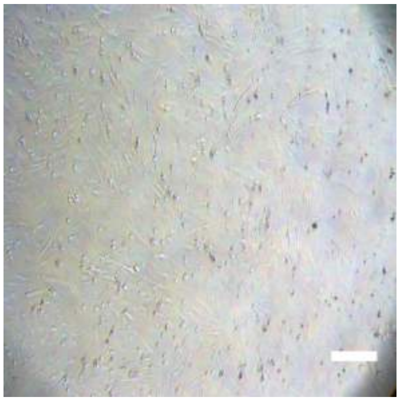

B. 5 days after virus

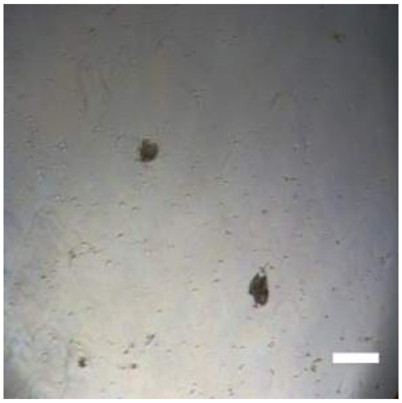

C. 21 days after virus

WSB/EiJ

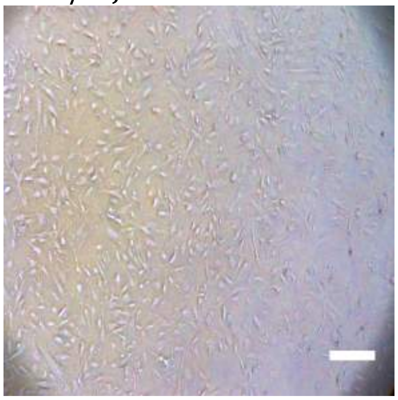

D. day before the virus

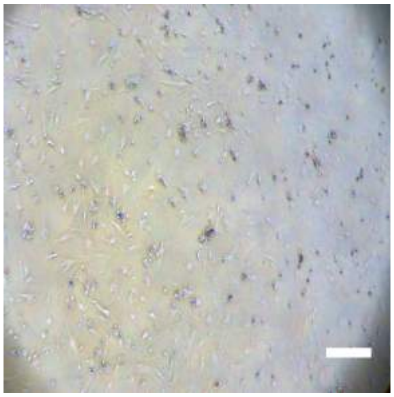

E. 5 days after virus

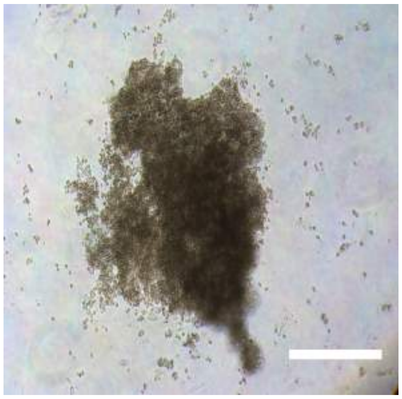

F. 18 days after virus

Figure 1: Serum free conditions during reprogramming leads to cell death
(A,B,D,E) Approximately five days after the addition of 2i in serum free conditions, cell death became obvious in both backgrounds. (C,F) By 18 – 21 days after the addition of 2i in serum free conditions all the cells were dead and detached from the plate. Some floating clumps were selected and placed on MEFs but did not expand. Scale bars are 400 μm.

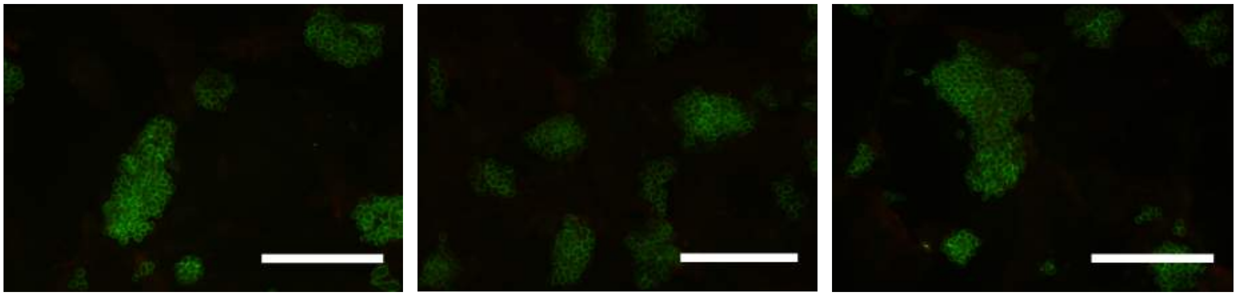

A. NOD/LtJ iPSC.2iS Male B. NOD/LtJ iPSC.2iS Female C. WSB/EiJ iPSC.2iS Male

Figure 2: NOD male and female iPSC grown in 2iS stain homogenously for PECAM1
The cells were stained for the ESC marker PECAM1 (green) and the EpiSC maker CD40 (red). There are no differences in cell surface staining between male and female NOD/ShiLtJ iPSC. Scale Bars are 200 μm.

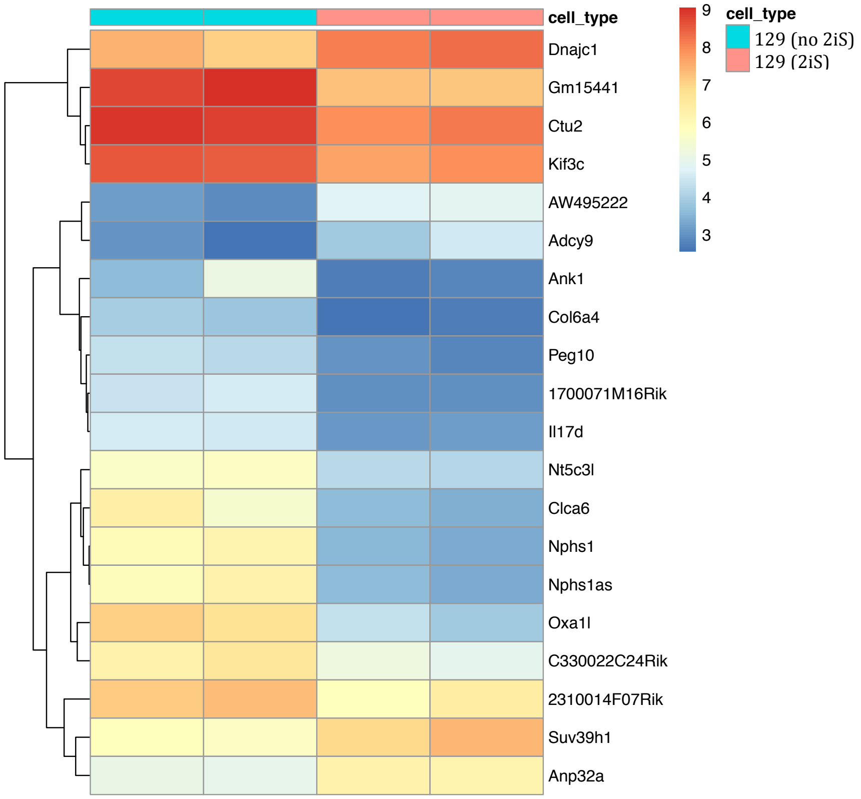

Figure 3: Difference between 129S1/SvImJ iPSC maintained in 2iS media versus ESC/LIF media

129S1/SvImJ male iPSC were derived and maintained in standard ESC/LIF media for 16 passages and then transferred and maintained in 2iS media for eight passages. Their transcriptional profiles were compared, revealing 39 differentially expressed genes, 20 of which are depicted here with varying degrees of expression. Cells maintained in 2iS media had reduced expression of *Ank1*, *Col18A1*, *NphS1*, and *I117D*, genes associated with cell death. Lower expression of *Kif3C*, *Basp1*, and *NphS1* in 2iS maintained cells compared to ESC/LIF maintained cells, is associated with reduced cytoskeleton organization.
